# Test and evaluation of driving comfort of rice combine harvester

**DOI:** 10.1371/journal.pone.0287138

**Published:** 2023-06-14

**Authors:** Ying Zhao, Jinyi Liu, Lina Ma, Jianfei Zhang, Caixue Zhan

**Affiliations:** 1 Nanjing Institute of Agricultural Mechanization, Ministry of Agriculture and Rural Affairs, Nanjing, China; 2 Mechanical and Electrical Engineering College, Hainan University, Haikou, China; 3 Huazhong Agricultural University & College of Engineering, Wuhan, China; China University of Mining and Technology, CHINA

## Abstract

During the field operation of rice combine harvester, the vibration generated by vibration component will only reduce mechanical reliability and yield, but also cause resonance in the human body, leading to a decrease in driving comfort and even damage to the driver’s health. To study the impact of combine harvester vibration on driving comfort, a certain type of tracked rice combine harvester was selected as the research object, and vibration tests were carried out based on vibration source analysis in the driving cab during field harvesting operations. The study showed that under the influence of field road conditions and crop flow, the operating speed of the engine, threshing rotor, stirrer, cutting blade, threshing cylinder, vibration sieve and conveyor were fluctuating, and their rotation and reciprocating motion would produce vibration excitation in the driving cab. A spectrum analysis was conducted on the acceleration signal of the driver’s cab, and it was found that vibration frequencies at three measuring points, namely the pedal, control lever, and seat, can reach up to 36.7~43.3 Hz. These frequencies can cause resonance in various parts of the driver’s body, such as the head and lower limbs, leading to symptoms such as dizziness, throat discomfort, leg pain, defecation anxiety, and frequent urination, and even affect vision of the driver. At the same time, a weighted root-mean-square acceleration evaluation method was used to evaluate the driving comfort of the harvester. The evaluation method showed that the vibration at the foot pedal (*A*_*w*1_ = 4.4 m/s^2^>2.5 m/s^2^) caused extreme discomfort, while the vibration at the seat (0.5 m/s^2^< *A*_*w*2_ = 0.67 m/s^2^<1.0 m/s^2^) and the control lever (0.5 m/s^2^< *A*_*w*3_ = 0.55 m/s^2^<1.0 m/s^2^) caused relatively less discomfort. This research can provide some reference for the optimization design of the joint harvester driver’s cab.

## 1. Introduction

As an extensively applied large-scale field harvesting machine, the combine harvester can not only significantly improve agricultural productivity and reduce labor intensity, but also has important significance for ensuring the grain harvest in China [1–4]. However, during operation of rice, wheat and oilseed harvesting machinery in the field, the machine body will produce large noise and strong vibration due to the impact of uneven road surface in the field and multi-source coupling vibration excitation of engines, cutters, vibration sieves, threshing cylinders and conveyors [5–9]. The body vibration will be transmitted to the human body through seats, pedals, joystick and other components, thus causing discomfort to the human body. Working in this environment for a long time will reduce the sensitivity of the driver, increase the threshold value of vibration sense, and cause bone joint damage, bone hyperplasia, muscle atrophy and other adverse effects, thereby threatening the driver’s health [10–13]. At the same time, poor vibration will not only reduce crop yield, but also harm the reliability of the machinery itself [8]. For machinery operating under vibration conditions for a long time, fasteners are easy to loosen, and mechanical structures or parts are more likely to have material fatigue and failure under the action of alternating force caused by vibration [14, 15]. When the natural frequency of the mechanical structure is consistent with the frequency of the external excitation, resonance will also occur, resulting in excessive deformation of the mechanical structure, which will further aggravate the driver’s discomfort [8, 16]. Lim et al. [17] used the principal component analysis (PCA) method to calculate the multiple response performance index of the cab suspension system vibration of commercial vehicles, and optimized the reliability based robust design. By integrating industrial design and ergonomics, Fan et al.; Prabhahar et al.; Barreto et al. [18–20] constructed the model of virtual prototype by the softwares Adams, ProE and CATIA, and did optimization design on the layout, vibration attenuation of driver’s cab. Jayabalan et al. [21] installed a new type of active controller in the seat suspension system to improve the ride comfort by controlling the damping force of the magnetic fluid damper. Sim et al; Picoral Filho et al.; Carletti et al. [22–24] studied the factors affecting driver’s comfort through the three methods of subjective tests, case study and triaxial evaluation. Bordignon et al. [11] introduced a new evaluation standard for driver’s seat pressure, and evaluated the comfort of agricultural tractor seats by comparing the comfort index CI under static and dynamic conditions. Yang et al. [25] proposed a fast evaluation approach for the upper limbs to improve the tractor cab with taking into consideration the ergonomics. Fernandez et al. [26] designed a three-point hitch (THP) to ensure better dynamic load distribution and ultimately improve operational performance, comfort, and safety. According to the operator’s heart rate, posture comfort, Cornell ergonomics and other evaluation factors, Jayasuriya et al. [27] designed a new type of damping tractor seat suspension system. The studies above generally adopted the methods of simulation optimization, mathematical model, experimental simulation and dynamic calculation to optimize the design of automobile or tractor cab, and there are few studies on the optimization design of harvester cab. Moreover, since harvesters are subject to multi-source coupling vibration excitation, and the field operation conditions are relatively complex, there is somewhat deviation between the theoretical calculation and simulation data and the data under the actual field operation conditions. Therefore, in this paper, by taking a type of domestic crawler-type rice combine harvester as the research object, under the field harvest condition, vibration tests were carried out on the pedal, joystick and seat of the harvester cab respectively. Then, based on the time-domain and frequency-domain analysis, the key excitation source and vibration frequency that cause driver vibration and the impact of the harvester cab vibration on the human body were determined. At the same time, 1/3 octave analysis method and effective value evaluation method for weighted acceleration were used to analyze and evaluate the driving comfort.

## 2. Materials and methods

### 2.1 Analysis on the main vibration sources of rice combine harvester

**Fig 1:**
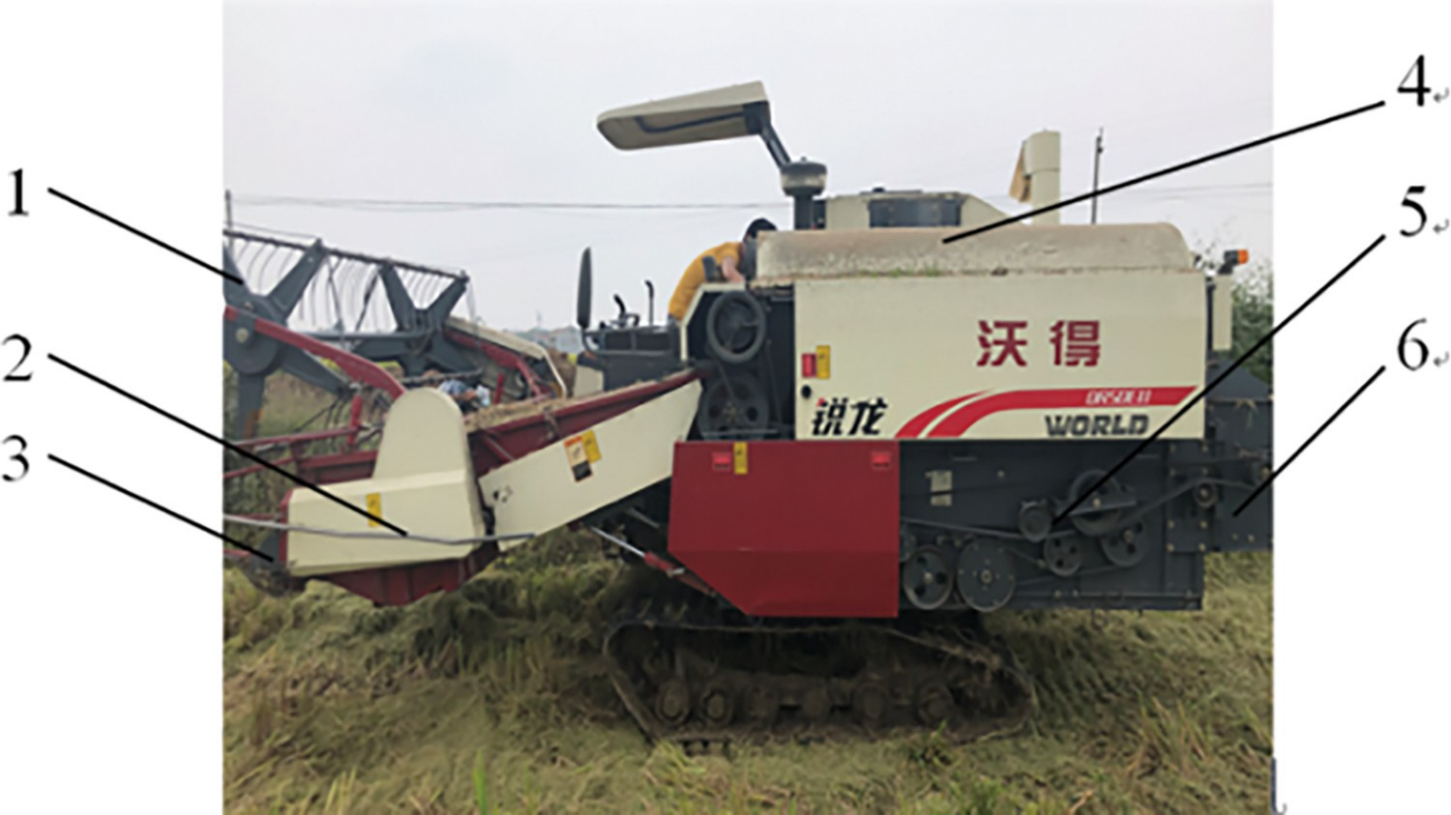
Overall structure of a crawler-type rice combine harvester in China. **Note:** 1- Reel 2-Stirrer 3-Cutting blade 4-Threshing cylinder 5-Vibration sieve 6-Conveyor.

**Fig 2:**
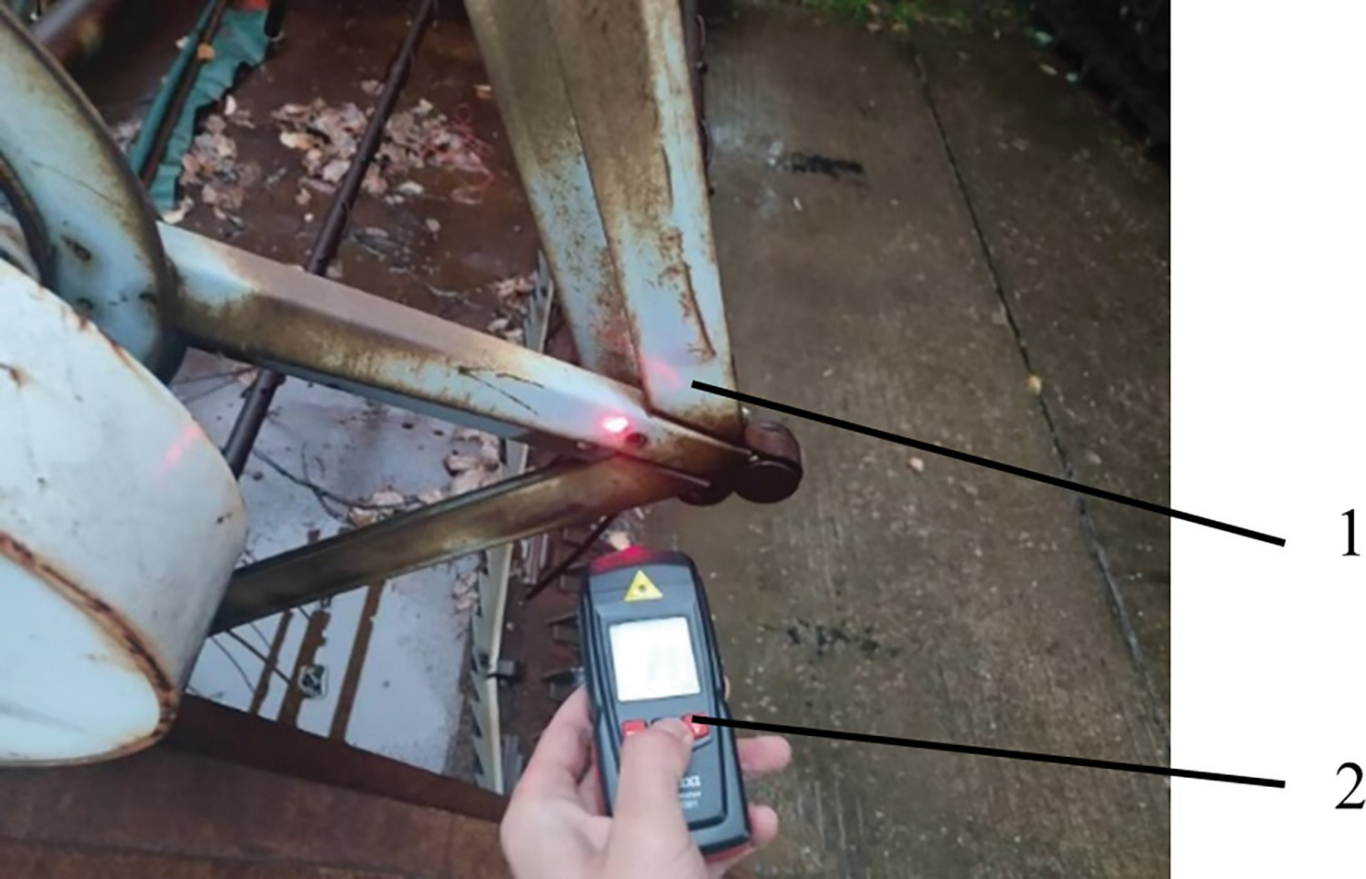
Photoelectric tachometer measures the rotational speed at the reel. **Note:** 1- Reel 2- Photoelectric tachometer.

A kind of crawler-type rice combine harvester made in China was selected in the analysis, and its overall structure is shown in Fig 1, and structural parameters are shown in Table 1. The vibration sources of the rice combine harvester mainly includes the engine, the stirrer, the vibration sieve, the cutting blades, the conveyor, the threshing cylinder, the reel and road surface excitation, which can cause vibration after the equipment is started. Under field operating conditions, the Shengli VC6234P type light electric tachometer was used to measure the working speed of the main components of the rice combine harvester. The working principle of this photoelectric tachometer adopts optical technology and non-contact measurement method. Reflective paper is pasted on the tested working parts (the test parts cannot be reflective objects). The instrument measures the reflective paper on the tested parts to obtain the working speed of the main working parts. Fig 2 shows the schematic diagram of the photoelectric tachometer measuring the rotational speed at the reel. Then, the theoretical vibration frequency of each working part is calculated by Eq (1), and the results are shown in Table 2. These vibrations will be transmitted to the cab and affect the driver’s driving comfort.

$$f=\frac{n}{60}$$ (1)

Where, *f* is vibration frequency, Hz; *n* is the measured speed of main components during field operation, r/min;

**Table 1:** Main structural parameters of the crawler-type rice combine harvester.

Main structural parameters	Data
Size (Length× Width ×Height)/ mm	5590×2670×2875
Machine weight/kg	3000
Engine type	4G33TC
Engine pattern	Vertical/In-line/Four-stroke
Minimum ground clearance/mm	320
Threshing method	Tooth-type threshing in longitudinal axial flow

**Table 2:** Main working parameters in field harvesting operation of the crawler-type rice combine harvester.

Name of components	Rotating speed/(r·min^-1^)	Theoretical vibration frequency/Hz
Engine	2200~2600	36.7~43.3
Stirrer	528.6~624.7	8.8~10.4
Vibration sieve	402.3~475.5	6.7~7.9
Drive shaft of the cutting blade	398.1~470.5	6.6~7.8
Conveyor	313.8~370.8	5.2~6.2
Threshing cylinder	95.6~113.0	1.6~1.9
Reel	46.7~55.1	0.8~0.9

### 2.2 Vibration testing scheme for the driver’s cab in field

The 1A314E type piezoelectric accelerometer was adopted to test the vibration status of the rice combine harvester in field harvesting operation. The Donghua DH5902 type dynamic signal acquisition instrument was adopted to acquire signals, and the main technical parameters of the equipment are shown in Table 3.

**Table 3:** Main technical parameters of the test data acquisition and analysis equipment.

Name of the equipment	Parameters		Values	
1A314E type piezoelectric accelerometer	Range/(m·s^-2^)			±500
	Axial sensitivity/(mV/g)			10
	Maximum horizontal sensitivity/%			<5
	Frequency response/Hz			0.5~7 000
	Resolution/(m·s^-2^)			0.004
DH5902 type dynamic signal acquisition instrument	Passage	32		
	Sampling bandwidth/kHz	100(16-bit)		
	Full-scale value/mV	±20 000		
	Degree of distortion/%	<0.5		

**Fig 3:**
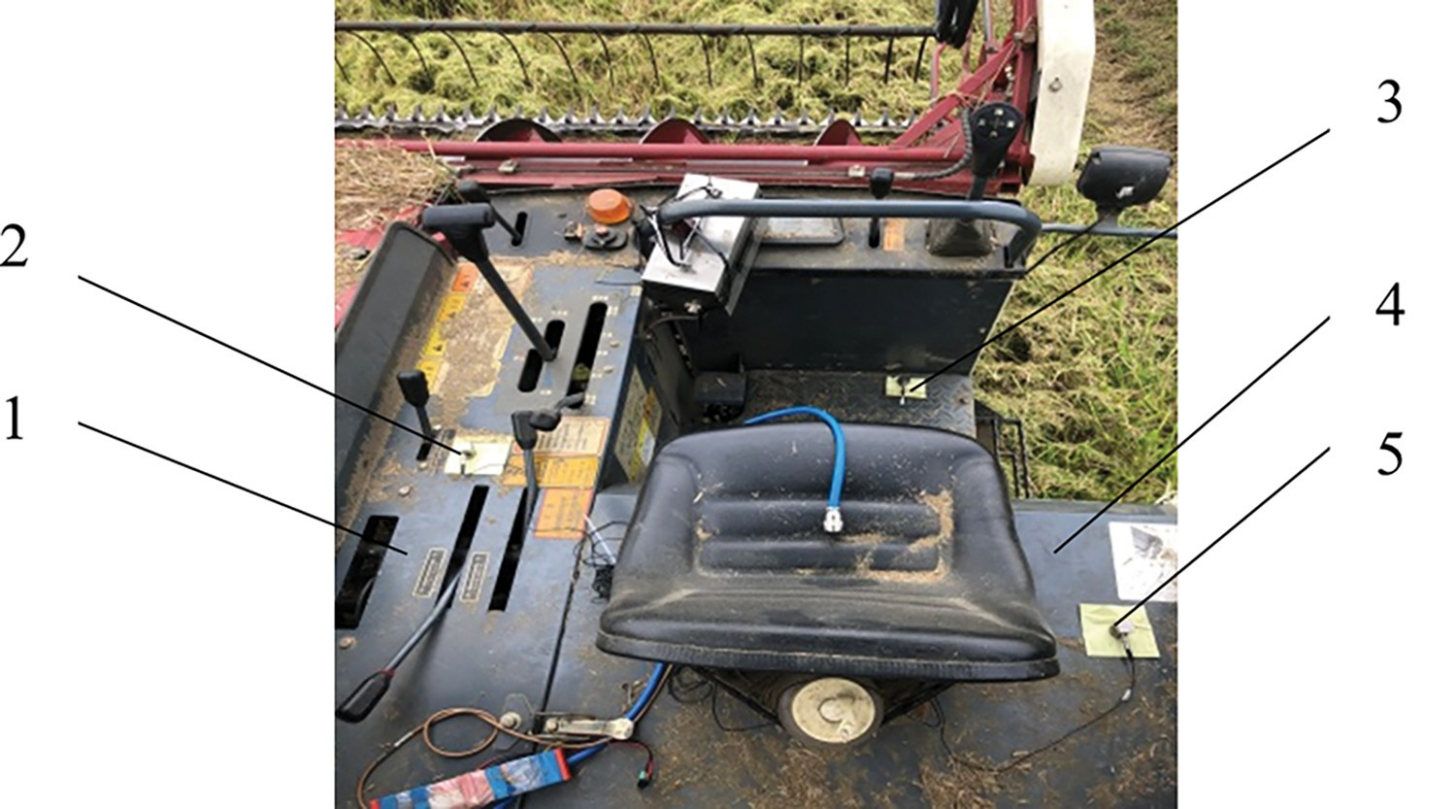
Schematic diagram of measuring points. **Note:** 1-Joystick installation platform 2-Measuring point at the joystick 3-Measuring point at the pedal 4-Seat installation base 5-Measuring point at the seat.

The vibration test was conducted in September 2020 in the rice demonstration field of Xiangyang City, Hubei Province, China. The vibration of the cab testbed of the rice combine harvester was tested under the condition of field harvest operation. The test measuring points were respectively arranged at the foot pedal (measuring point 1), joystick (measuring point 2) and seat (measuring point 3), as shown in Fig 3. The direction of X, Y, Z channels in the sensor correspond to the forward direction, lateral direction and vertical direction of the harvester respectively. In the DHDAS dynamic signal analysis system, set the instrument model as DH5902, the sampling frequency fs as 200 Hz (sampling interval at 1 ms), and the sampling time as 120 s.

## 3. Results and discussion

### 3.1 Time-domain analysis

The acceleration signals of the three measuring points of the driving platform under field harvesting operation conditions were processed and analyzed in time domain. The root mean square of vibration acceleration of measuring points 1, 2 and 3 in X, Y and Z directions are shown in Table 4.

**Table 4:** Root mean square of acceleration in different directions at each measuring point (m/s^2^).

Measuring point	Root mean square of acceleration (m/s^2^)		
	*X*	*Y*	*Z*
1	19.96	59.54	16.00
2	6.06	4.54	5.07
3	5.52	2.85	2.53

It can be seen from Table 4 that the foot pedal (measuring point 1) is close to the engine, transmission parts and working parts, without any damping device. Therefore, the root mean square value of vibration acceleration of the foot pedal in the X, Y and Z directions is larger than that at the joystick and the seat. While for the joystick and the seat, the additional joystick installation platform and seat installation support were added for buffering and vibration attenuation to some extent, therefore, the root mean square value of acceleration at the joystick and the seat was relatively small. The foot pedal is in the Y direction, that is, in the left and right directions, and the root mean square of the vibration acceleration was 59.54 m/s2. The reason is that, a header was installed in front of the foot pedal, and the header cutter did reciprocating movement in the left and right directions, causing severe vibration in the Y direction at the foot pedal.

### 3.2 Frequency-domain analysis

After processing and analyzing the acceleration signals of the three measuring points under the field harvesting condition, the vibration frequency and amplitude of the three measuring points were obtained, as shown in Table 5.

**Table 5:** Vibration frequency and amplitude of the first six orders at each measuring point.

Measuring points	Series	*X*		*Y*		*Z*	
		Vibration amplitude	Vibration frequency	Vibration amplitude	Vibration frequency	Vibration amplitude	Vibration frequency
		Amp/(m/s^2^)	Freq/Hz	Amp/(m/s^2^)	Freq/Hz	Amp/(m/s^2^)	Freq/Hz
Measuring point 1	1	2.21	3.52	5.62	2.64	1.86	3.18
	2	1.65	1.82	4.93	4.34	1.43	5.11
	3	1.47	9.60	2.91	7.46	1.17	9.60
	4	1.24	12.82	2.50	10.55	0.97	19.68
	5	0.98	24.71	1.89	18.46	0.88	24.71
	6	0.83	37.44	1.45	22.75	0.84	30.69
Measuring point 2	1	0.60	9.23	0.92	0.66	0.45	3.48
	2	0.54	14.37	0.63	1.23	0.36	4.32
	3	0.45	5.64	0.36	3.73	0.32	11.36
	4	0.43	18.57	0.30	18.09	0.25	15.21
	5	0.37	25.28	0.26	21.25	0.24	20.62
	6	0.35	29.66	0.25	33.23	0.22	62.85
Measuring point 3	1	0.74	12.32	0.29	15.55	0.26	26.32
	2	0.68	14.37	0.28	3.41	0.25	72.11
	3	0.64	4.59	0.27	24.01	0.23	62.92
	4	0.58	27.87	0.20	32.92	0.21	20.6
	5	0.52	18.87	0.17	40.01	0.20	79.15
	6	0.35	42.28	0.16	57.15	0.17	11.37

It can be obtained by comparing Tables 2 and 5 that

(1) 37.44, 40.01 and 42.28Hz are the sixth order vibration frequency in the X direction at measuring point 1, the sixth order vibration frequency in X direction at measuring point 3 and the fifth order vibration frequency in Y direction at measuring point 3, respectively, which are close to the theoretical vibration frequency of the engine (36.7~43.3 Hz). Therefore, the main excitation source causing the vibration in the front and rear directions at the foot pedal and the horizontal direction at the seat is the engine.

(2) 9.60, 10.55, 9.67, 9.23, 11.36 and 11.37Hz belong to the range of the theoretical vibration frequency (8.8~10.4Hz) of the auger, so the main excitation sources in X, Y, Z directions at measuring point 1, X, Z directions at measuring point 2 and Z direction at measuring point 3 were the vibration at the auger.

(3) The fourth order frequency in Y direction at measuring point 1 was 7.46Hz, which is close to the theoretical frequency range of vibration sieve (6.7~7.9Hz) and cutting blade (6.6~7.8Hz), so this direction was also affected by the reciprocating vibration excitation of the vibration sieve and the cutting blade.

(4) The frequency in Y direction of measuring point 1 and in X direction of measuring point 2 were 5.11Hz and 5.64Hz respectively, which are within the theoretical vibration range (5.2~6.2Hz) of the conveyor, so the vibration frequency was also affected by the conveyor.

(5) The second order frequency in X direction at measuring point 1 was 1.82Hz, which is close to the theoretical vibration frequency of the threshing cylinder (1.6~1.9Hz). Therefore, threshing cylinder is one of the excitation sources of vibration here.

(6) The first order frequency in Y direction at measuring point 2 was 0.66Hz, which is close to the theoretical vibration frequency of the reel (0.8~0.9Hz). Therefore, the reel is one of the excitation sources causing the vibration here.

Therefore, when the harvester is operating in the field, the rotation and reciprocating motion of the engine, the reel, the auger, the cutting blade, the threshing cylinder, the vibration sieve, the conveyor will generate vibration excitation to the cab, and the vibration will be transmitted to the human body through the seat, the pedal, the joystick and other parts. The human body is composed of elastic tissues. When the human body is subjected to external vibration, its reaction mode is similar to an elastic system. Human organs and external vibration will produce resonance. When the external vibration frequency force falls within the resonance range, resonance will occur, causing damage to human parts or organs and affecting human health [23]. It can be seen from Table 6 that the human body parts or organs damaged by different vibration frequencies are different. The vibration frequency of the engine is within the resonance frequency of the eyes, which will lead to eye congestion, vision loss and other symptoms. The vibration frequency of the stirrer, the vibration sieve, the cutting blade and the conveyor is just within the resonance frequency range of human head and lower limbs, which causes resonance of head and lower limbs, and seriously causes dizziness, throat discomfort, lower limb pain, defecation anxiety and frequent urination. The vibration frequency of the stirrer is also within the resonant frequency range of the abdominal cavity and spine of the human body, which will lead to stomach pain, dizziness, nausea and other discomfort. The vibration frequency of vibration sieve, drive shaft of the cutting blade and the conveyor is within the range of chest resonance frequency, thus it will cause chest pain, dyspnea, rapid heart rate, accelerated muscle contraction and other symptoms.

**Table 6:** Resonance frequency range of body parts or organs (Hz).

Resonance frequency range/Hz	Human body parts or organs
30~80	Eyes
2~30	Head and lower limbs
10~12	Abdominal cavity, spine
4~8	Chest

### 3.3 Evaluation of human driving comfort in field operation

With reference to the national standard GB/T8421-2000, the international standard ISO2631-1-1997 (E) and the national standard GB/T13,876–2007, the driving comfort of rice harvester during field harvesting operation was analyzed and evaluated by using the weighted acceleration root mean square value. Considering the interference effect of vibration of different frequency bands on human perception when broadband random vibration occurs, the effective value of vibration acceleration of other frequency bands beyond the most sensitive frequency range of human body is frequency weighted, which is equivalent to the effective value of vibration acceleration in the most sensitive frequency range, and this is called the effective value of weighted acceleration, which is expressed in *a*_*wj*_. The relationship between *a*_*wj*_ and *a*_*fj*_ of 1/3 original octave is:

$$a_{wj}=W_{fj}a_{fj}$$ (2)

Where, *a*_*wj*_ is the frequency weighting function; *a*_*fj*_ is the root mean square value of vibration acceleration at the center frequency of the *j*-th frequency band in 1/3 octave; *W*_*fj*_ is the corresponding weighting factor (referring to GB/T8421-2000)

For vertical vibration,

$$W_{fj}=\{\begin{array}{l} 0.5f_{cj}^{\frac{1}{2}}(1<f_{cj}\leq 8)\\ 1(4<f_{cj}\leq 8)\\ \frac{8}{f_{cj}}(8<f_{cj}) \end{array}$$ (3)

For horizontal vibration,

$$W_{fj}=\{\begin{array}{l} \frac{2}{f_{cj}}(2<f_{cj})\\ 1(1<f_{cj}\leq 2) \end{array}$$ (4)

According to the principle of energy addition, within the frequency range of 1~80 Hz, calculate the root mean square value of vibration acceleration at the 1/3 octave frequency band of each measuring point in the *X*, *Y* and *Z* directions. The measured root mean square value of vibration acceleration at different frequency bands in the X direction at measuring point 1 is shown in Table 7. Then calculate the effective value of the total weighted acceleration aw according to the *a*_*wj*_ of each frequency band, and evaluate it with *a*_*w*_.

**Table 7:** Root mean square of vibration acceleration of the 1/3 octave bands of measuring point 1 in X direction.

No.	Center frequency/Hz	Root mean square of acceleration (m/s^2^)	No.	Center frequency/Hz	Root mean square of acceleration (m/s^2^)
1	1	11.13	11	10.00	4.16
2	1.25	10.15	12	12.50	3.21
3	1.6	9.21	13	16.00	2.12
4	2.00	8.23	14	20.00	1.18
5	2.5	7.25	15	25.00	0.18
6	3.15	6.18	16	31.50	0.88
7	4.00	5.10	17	40.00	1.86
8	5.00	4.16	18	50.00	5.83
9	6.30	3.21	19	63.00	6.84
10	8.00	2.12	20	80.00	7.86

The measured root mean square values of vibration acceleration in 20 different frequency bands of 1/3 octave were weighted and amended with different weighting factors according to the different degrees of influence of the center frequency on the human’s vibration sensation, and then the effective value of the total weighted acceleration is calculated as:

$$a_{w}=\sqrt{\sum_{j=1}^{20}a^{2}{}_{fj}W^{2}{}_{fj}}$$ (5)

Where, *a*_*w*_ is effective value of total weighted acceleration.

When the vibrations of the seat surface in the three axial directions, *X*, *Y* and *Z* are considered at the same time, the root mean square value of the combined total weighted acceleration in the three directions is calculated according to Eq (6).

$$A_{w}=\sqrt{{\left(1.4a_{xw}\right)}^{2}+{\left(1.4a_{yw}\right)}^{2}+a_{zw}{}^{2}}$$ (6)

Where *a*_*xw*_, *a*_*yw*_ and *a*_*zw*_ represent the root mean square values of joint weighted accelerations in X, Y and Z directions, m/s^2^.

The calculated root mean square values of the joint weighted accelerations at the three measuring points of the pedal, joystick and the seat are *A*_*w*1_ = 4.40 m/s^2^, *A*_*w*2_ = 0.67 m/s^2^, and *A*_*w*3_ = 0.55 m/s^2^ respectively. It can be obtained by referring to Table 8 (national standard ISO2631:1997 (C)) that, the vibration generated at the pedal makes people feel extremely uncomfortable, and the vibration at the seat and joystick makes people feel uncomfortable.

**Table 8:** The relationship between *A*_*w*_ and human feelings.

Root mean square of joint weighted acceleration (m/s^2^)	Human feelings
<0.315	No discomfort
0.315~0.63	A little discomfort
0.5~1.0	Some discomfort
0.8~1.6	Much discomfort
1.25~2.5	Very much discomfort
>2.5	Extreme discomfort

## 4. Conclusions

(1) The pedal at measuring point 1 is close to the engine, transmission parts and working parts, and there is no damping device. Therefore, the root mean square value of vibration acceleration of the foot pedal in the X, Y and Z directions is larger than that at the joystick and the seat.

(2) In the field harvest operation, affected by the field road surface and crop flow, the operating speeds of the engine, reel, stirrer, cutting blade, threshing cylinder, vibration sieve, conveyor and other working parts are fluctuating values. The rotation and reciprocating motion of these components will bring about vibration excitation to the driver’s cab.

(3) Under the field harvest operation condition, the vibration of working parts will be transmitted to the human body through seat, pedal, joystick and other parts. The vibration frequency of these working parts will cause resonance in the driver’s head, lower limbs, eyes, abdominal cavity, spine and chest. Long term operation under this condition will cause the driver to feel dizzy, chest pain, muscle contraction, lower limb pain, stool anxiety and urine frequency.

(4) The root mean square values of weighted acceleration were used to evaluate the driving comfort of the harvester in vibration. The vibration generated at the foot pedal (*A*_w1_ = 4.4 m/s^2^ > 2.5 m/s^2^) made people feel extreme discomfort, while the vibration at the seat (0.5 m/s^2^<*A*_w2_ = 0.67 m/s^2^<1.0 m/s^2^) and the vibration at the joystick (0.5 m/s^2^< Aw3 = 0.55 m/s^2^<1.0 m/s^2^) made people feel some discomfort.
